# Effectiveness of three nasoenteral tube securement technologies in critically ill adults: A pragmatic cluster-randomized crossover trial

**DOI:** 10.1016/j.ijnsa.2026.100729

**Published:** 2026-10-27

**Authors:** Alan Maique Ribeiro Fernandes da Costa, Oleci Pereira Frota, Marcos Antonio Ferreira Junior, Daniele Vieira Dantas, Maria Luiza Berti de Oliveira, Milena Agnelo Santos Chaves, Danielle Neris Ferreira, Elaine Aparecida Rocha Domingues

**Affiliations:** a School of Nursing, Universidade Federal de Mato Grosso do Sul, Av. Costa e Silva s/n. Cidade Universitária, Bloco 12 -CP. 649, Campo Grande, Mato Grosso do Sul, Brazil; b Department of Nursing, Universidade Federal do Rio Grande do Norte, Rua General Cordeiro de Faria, S/N, CP 1524 - Petrópolis, Natal, Rio Grande do Norte, Brazil; c Hospital Universitário Maria Aparecida Pedrossian, Campo Grande, Brazil. Av. Sen. Filinto Müller, 355 - Vila Ipiranga, Campo Grande, Mato Grosso do Sul, Brazil; d Instituto Federal do Norte de Minas Gerais, Rodovia BR 367, Km 07, s/n – Zona Rural, Almenara, Minas Gerais, Brazil

**Keywords:** Nursing, Intensive care units, Intubation, Gastrointestinal, Adhesives, Enteral nutrition, Pragmatic clinical trial

## Abstract

**Background:**

Nasoenteral tube stability is essential for the success of enteral nutritional therapy in critically ill patients. However, comparative evidence regarding the effectiveness and safety of available securement technologies remains limited, particularly concerning the balance between tube stability and preservation of skin and mucosal integrity.

**Objective:**

To compare the effectiveness and safety of three nasoenteral tube securement technologies in critically ill patients.

**Design:**

A pragmatic, cluster-randomized, crossover, controlled, superiority, triple-blind clinical trial.

**Settings:**

The study was conducted in intensive care units at three large Brazilian hospitals.

**Participants:**

A total of 217 patients were included and received one of three securement strategies according to their intensive care unit-period allocation: manufactured securement using acrylate adhesive tape (control; *n*=75), a dedicated device with acrylate adhesive (*n*=72), or a dedicated hydrocolloid-based device (*n*=70).

**Methods:**

The participants were prospectively monitored during nasoenteral tube use. Outcomes included tube displacement and inadvertent removal, securement durability, device-related skin and mucosal injuries, and interruption of enteral nutritional therapy. Comparisons between groups were performed using generalized linear mixed models and survival analysis.

**Results:**

No statistically significant differences were observed among the groups regarding nasoenteral tube displacement or inadvertent removal. The hydrocolloid group showed a higher risk of securement failure compared to the control group (hazard ratio=1.93; 95% confidence interval: 1.28–2.90; *p*=0.002) but a lower incidence of medical adhesive-related skin injury (7.1%) than the control group (34.7%) and the acrylate group (38.9%). The lower incidence of medical adhesive-related skin injury in the hydrocolloid group compared with the control group corresponded to an absolute risk reduction of 28% and a number needed to treat of 3.6. There were no significant differences among the groups regarding mucosal membrane injury or the duration of enteral nutrition therapy interruption.

**Conclusions:**

Dedicated securement technologies did not demonstrate superiority over improvised securement methods in preventing displacement or inadvertent removal of the enteral feeding tube. However, the hydrocolloid-based device reduced the medical adhesive-related skin injury incidence, despite showing lower securement durability. These findings highlight a trade-off between mechanical stability and the preservation of skin integrity, reinforcing that the choice of securement technology should be individualized, taking into account the patient’s risk profile and the care setting.

**Registration:**

The trial was registered in the Brazilian Registry of Clinical Trials (ReBEC; RBR-10hr8nzd).

**What is already known**

• The method used to secure a nasoenteral tube influences device stability, continuity of enteral nutrition therapy, and the occurrence of adverse events in critically ill patients.
• Dedicated securement devices and manufactured adhesive tape methods are widely used; however, robust comparative evidence on their effectiveness remains limited.
• There is no established consensus on how to balance securement durability with preservation of skin integrity when selecting a securement strategy.

**What this paper adds**

• Dedicated securement devices were not superior to manufactured securement methods in preventing nasoenteral tube displacement or inadvertent removal in critically ill adults.
• When performed using a standardized technique, manufactured securement achieved outcomes comparable to dedicated securement devices, highlighting the role of standardized nursing practice.
• The hydrocolloid-based device reduced medical adhesive-related skin injury but showed lower securement durability.

## 1 Introduction

Enteral nutrition therapy is an essential component of critical care, and its effectiveness depends on the secure maintenance of the nasoenteral tube. Unplanned tube dislodgement compromises the continuity of nutritional therapy, increases the need for tube replacement or repositioning, and exposes patients to potentially preventable adverse events (Ribeiro et al., 2025). Securement strategy is one of the potentially modifiable factors influencing nasoenteral tube stability. Clinical practice employs both manufactured securement methods and dedicated securement devices. A manufactured securement method refers to a fixation method manually constructed by healthcare professionals from cut strips of adhesive tape to secure the nasoenteral tube, whereas a dedicated securement device refers to an industrially manufactured product specifically designed for this purpose. However, there is no consensus regarding which strategy offers superior performance. Beyond tube stability, the securement strategy may also affect skin and nasal mucosa integrity, thereby contributing to adverse outcomes, such as displacement, inadvertent removal, or skin or mucosal injury (Anziliero, Beghetto, 2022; Motta et al., 2021).

The reported frequency of these adverse events varies considerably across the literature. Researchers have reported nasoenteral tube displacement incidence of up to 62%, inadvertent removal rates ranging from 53.8% to 70.1%, and medical adhesive-related skin injury in up to 42% of patients exposed to medical adhesives (Anziliero; Beghetto, 2022; Frota et al., 2025). However, these estimates derive from heterogeneous studies in terms of design, operational definitions, and evaluated outcomes, making comparisons between securement technologies difficult and reducing the applicability of the results to clinical practice.

Researchers have predominantly focused on preventing tube loss or evaluated specific technologies in isolation (Aeberhardt; Bains; Desai, 2022; Anziliero, F., Beghetto). Researchers have directly compared manufactured securement methods and dedicated devices using robust experimental designs and simultaneously evaluating outcomes of mechanical stability, securement durability, and cutaneous-mucosal safety. Thus, it remains uncertain which technology offers the best balance between maintaining nasoenteral tube and preserving tissue integrity in critically ill patients. Given this gap, we aimed to compare the effectiveness and safety of three nasoenteral tube securement technologies in critically ill patients.

## 2 Methods

### 2.1 Study design

This was a pragmatic, cluster-randomized, controlled, superiority, triple-blind (participants, outcome assessors, and statisticians) clinical trial with a crossover design, conducted across three groups of nasoenteral tube securement technologies.

### 2.2 Study setting and participant recruitment

The study was conducted in the adult intensive care units of three large hospitals in Brazil, totaling 35 medical and surgical beds. Eligible patients were consecutively recruited between January and November 2025. Following screening, eligible participants received the securement technology assigned to their intensive care unit during the corresponding study period.

### 2.3 Eligibility criteria

All patients admitted to the intensive care unit were included, aged 18 years or over, with clinical indication for nasoenteral tube use and absence of previous injury to the skin or nasal mucosa at the securement site at the inclusion time. Those with known allergies or hypersensitivity to securement device materials were excluded, along with: systemic dermatological diseases that compromised skin integrity (such as Stevens-Johnson syndrome, toxic epidermal necrolysis, or epidermoclysis bullosa); structural skin abnormalities at the attachment site (for example, extensive scars or warts); or if the securement technology was used for less than 24 hours.

### 2.4 Sample size

The sample size was estimated for a cluster-randomized clinical trial comparing multiple proportions using the ‘n4props’ function from the ‘CRTSize’ package in R software. Expected success proportions of .80 in the experimental group and 0.40 in the control group were assumed, along with an intraclass correlation coefficient of .05, an average cluster size of 20 participants, a significance level of 5%, and a statistical power of 80%, incorporating an effect adjustment design. Based on these parameters, the calculation indicated a minimum requirement of 180 patients, distributed across three clusters over three sequential periods, totaling 60 patients per cluster.

### 2.5 Randomization and blinding

Randomization was performed at the cluster level: intensive care units. The three participating intensive care units were allocated a unique sequence of exposure to the three securement technologies, so that each intensive care unit applied all interventions over consecutive periods (Fig. 1). The sequence was generated by automatic randomization on the website randomization.com, in a single block, by an independent external researcher.

**Fig. 1:**
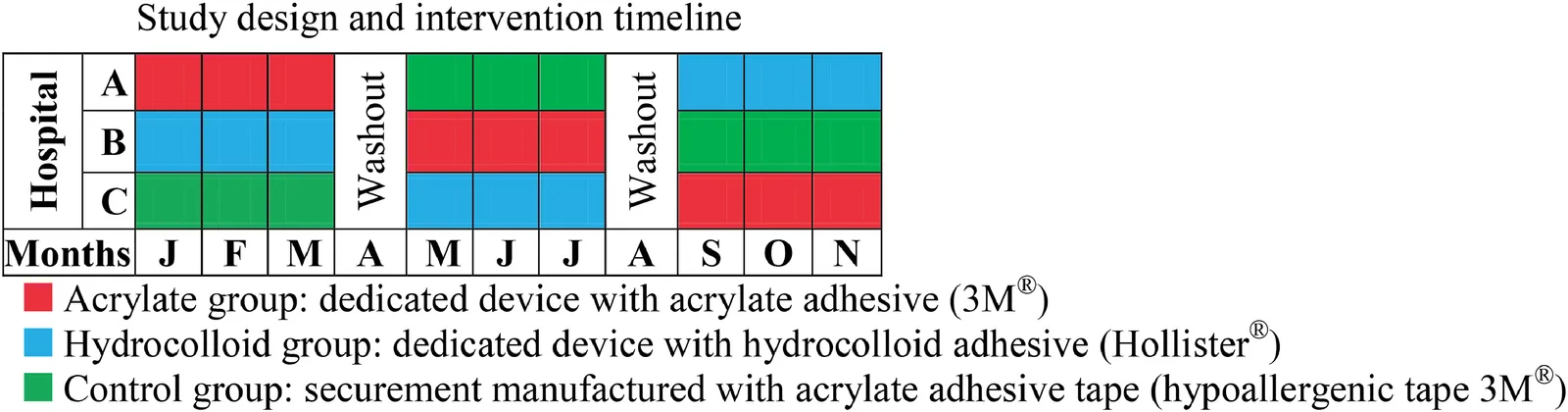
Study design with intervention timeline for nasoenteric tube securement.

Participants were blinded by not being informed whether the securement technology was standard or experimental. Outcome assessors did not participate in field data collection and independently evaluated photographs to determine the occurrence and classification of skin injuries. Objective outcomes, including securement detachment, tube dislodgement, inadvertent tube removal, and securement durability, were assessed prospectively by the field researchers according to predefined criteria. For statistical analysis, the three securement technologies were coded as A, B, and C, without identifying the technology or whether it belonged to the control or experimental group.

### 2.6 Intervention and control groups

Three nasoenteral tube securement technologies were compared (Fig. 1). Scheduled replacement followed the manufacturers’ recommendations and, therefore, differed between strategies: every 24 hours in the control and acrylate groups and every 7 days in the hydrocolloid group, or earlier when indicated by loss of adhesion, humidity, dirt, discomfort or compromised tube stability. The application, inspection, maintenance and replacement of technologies followed standard operating procedures prepared based on the recommendations of the respective manufacturers. Considering the recognized heterogeneity of manufactured securement techniques described in the literature and observed in clinical practice, a specific standard operating procedure was developed and previously validated for the control group through a methodological study using the Delphi technique and content validity index ≥0.80 (Vilalba, 2025) to standardize the intervention.

The acrylate group used the 3M® nasoenteral tube securement (3M do Brasil Ltda), composed of an acrylate adhesive base and a specific stabilization system for a nasoenteral tube. The hydrocolloid group used the Feeding Tube Attachment Device – FTAD (Hollister Inc., Libertyville, IL, USA), consisting of a hydrocolloid adhesive base and a specific system for stabilizing the nasoenteral tube. The control group used securement manufactured with hypoallergenic microporous acrylate adhesive tape (3M), applied manually according to a validated technique (Vilalba, 2025). The three securement technologies evaluated are illustrated in Supplementary Material, Figure S1.

### 2.7 Data collection procedures

Data were collected prospectively by seven field researchers previously trained and calibrated by the main researcher and a stoma nurse. The training covered the study protocols, the application of securement technologies, the medical adhesive-related skin injury, medical device-related pressure injuries, and mucosal membrane pressure injury diagnostic criteria, as well as the use of collection instruments. Calibration was conducted sequentially in a clinical simulation setting and at the bedside in the participating intensive care units, using structured checklists based on the study procedures and predefined diagnostic criteria. Agreement with the reference assessment was evaluated using Cohen’s kappa. All seven field researchers achieved a kappa coefficient >0.80 in both settings before beginning prospective data collection.

After inclusion, the securement technology normally used was replaced by that corresponding to the randomized group. Upon admission, sociodemographic and clinical variables were collected, and the Braden, Richmond Agitation-Sedation Scale, Glasgow, and Nutrition Risk in Critically III (NUTRIC) scales were applied when this information was not available in the medical record. Participants were monitored daily until definitive removal of the nasoenteral tube, discharge from the intensive care unit, death, or end of follow-up, with clinical variables and outcomes previously defined in the study protocol being recorded.

Suspicious lesions were photographed using a standardized protocol that preserved anonymity and independently assessed by two blinded enterostomal therapy nurses using National Pressure Injury Advisory Panel criteria (NPIAP, 2019) and the international medical adhesive-related skin injury consensus (McNichol et al., 2013). Disagreements were resolved by consensus. After diagnosis, the enterostomal therapy team was notified, and the securement site or nostril was rotated when feasible according to lesion type.

Episodes of partial or complete detachment were recorded, including the type of adhesive failure and the time elapsed since the last change; the securement device was immediately replaced in accordance with the group’s protocol. In cases of nasoenteral tube displacement or inadvertent removal, the event was documented, and the clinical care team was notified to confirm tube placement radiologically and to reposition the tube when indicated.

Interruptions to enteral nutrition therapy were measured in hours, representing the interval between the identification of nasoenteral tube displacement or inadvertent removal and resumption of the diet following radiological confirmation of tube placement. Interruptions resulting from medical exams, surgical procedures, patient transport, or other clinical indications were excluded.

### 2.8 Outcomes

The primary outcome was nasoenteral tube displacement, and secondary outcomes included inadvertent nasoenteral tube removal, durability of the securement technology, securement failure (partial or complete detachment), medical adhesive-related skin injury, medical device-related pressure injuries, mucosal membrane pressure injury, and duration of enteral nutrition interruption.

### 2.9 Ethical considerations

The study was approved by the research ethics committees of the participating institutions. Eligible participants with preserved autonomy were invited to participate and signed the informed consent form. In cases of impaired autonomy, the legal guardian was consulted for consent. The study was submitted to the Brazilian Registry of Clinical Trials with the number RBR-10hr8nzd. De-identified individual participant data, including the data dictionary, the statistical analysis code, and other study-related materials are available in the Supplementary Material.

### 2.10 Statistical analysis

Continuous variables were described by mean and standard deviation or median and interquartile range, depending on the distribution, and categorical variables by absolute and relative frequencies. Unadjusted comparisons used the Kruskal-Wallis and Pearson chi-squared tests.

Efficacy and safety outcomes were analyzed using generalized linear mixed models (GLMM), considering patients nested in hospitals, with group and period as fixed effects and hospital as a random effect. A binomial distribution with a logit link function was used for binary outcomes and Gaussian distribution for continuous outcomes. Effect measures were estimated based on marginal risks adjusted by marginal standardization and expressed as relative risk (RR), absolute risk reduction (ARR), number needed to treat or cause harm (NNT/NNH) and respective 95% confidence intervals (95% CI). The adjusted difference between means was estimated for continuous outcomes.

Time to nasoenteral tube displacement was analyzed using Kaplan-Meier curves, the log-rank test, and Cox regression with hospital-level frailty, adjusted for study period. Definitive tube removal, intensive care unit discharge, death, transfer, or end of follow-up were censored. Analyses were stratified when group-by-period interaction was detected. A 5% significance level and 95% CI were used. Multiple comparisons used Dunn’s test for continuous variables and Bonferroni correction for categorical and survival outcomes. Analyses were performed in R 4.3.2.

## 3 Results

### 3.1 Baseline characteristics

A total of 257 patients were assessed for eligibility, of whom 239 met the eligibility criteria and were randomized. After randomization, nine participants withdrew consent and 13 did not complete the minimum 24-hour exposure required for outcome assessment because of death, intensive care unit discharge, or transfer. Therefore, 217 participants were analyzed: Control (*n*=75), Acrylate (*n*=72), and Hydrocolloid (*n*=70) groups (Fig. 2). All participants received standard care according to institutional protocols, in addition to securement-specific care defined for each study group in the standard operating procedures.

**Fig. 2:**
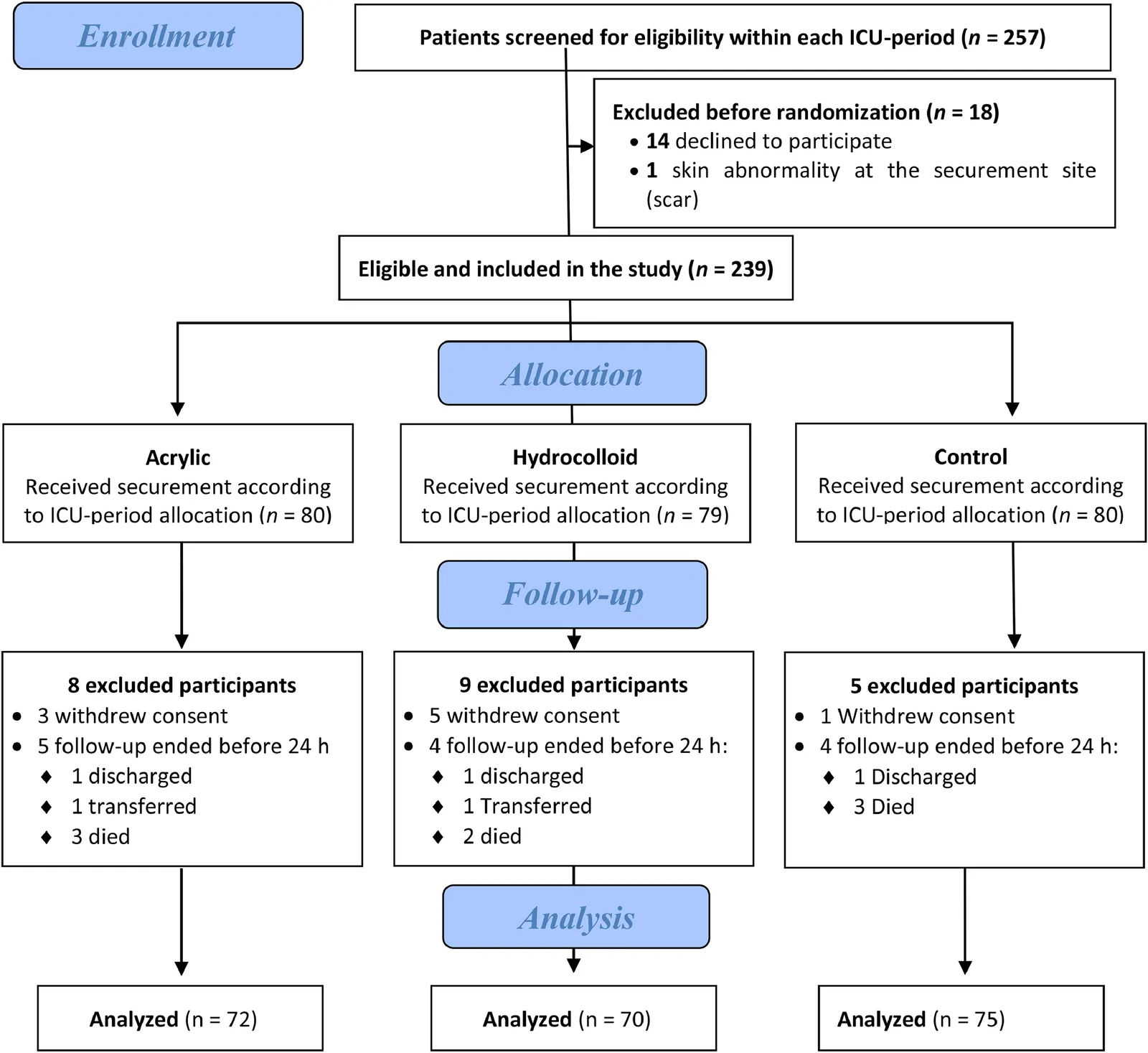
CONSORT flow diagram.

The sample was predominantly male, middle-aged, with white or brown skin color, and normal skin type. The majority had comorbidities, required mechanical ventilation, and had low mean Braden Scale and NUTRIC scores. There were no statistically-significant differences for most baseline characteristics regarding homogeneity between groups. Statistically-significant differences were observed between the groups for fever, length of corticosteroid use, Richmond Agitation-Sedation Scale score and reason for leaving the study (Table 1).

**Table 1:** Clinical and sociodemographic characteristics and homogeneity test between groups based on different types of nasoenteric tube securement.

Variables	Total (*n*=217) M±SD or n (%)	Group M±SD or *n* (%)			p*
		Hydrocolloid (*n*=70)	Acrylate (*n*=72)	Control (*n*=75)	
**Sex**					0.508
Female	92 (42.4)	33 (47.1)	27 (37.5)	32 (42.7)	
Male	125 (57.6)	37 (52.9)	45 (62.5)	43 (57.3)	
**Age** (years)	55.1±18.4	55.2±18.4	56.2±18.4	53.9±18.6	0.840
**Race/Skin color**					0.118
Yellow	02 (0.9)	00 (0.0)	02 (2.8)	00 (0.0)	
White	90 (41.5)	31 (44.3)	28 (38.9)	31 (41.3)	
Brown	91 (41.9)	34 (48.6)	28 (38.9)	29 (38.7)	
Black	34 (15.7)	05 (7.1)	14 (19.4)	15 (20.0)	
**Skin type**					0.078
Mixed	13 (6.0)	03 (4.3)	01 (1.4)	09 (12)	
Normal	150 (69.1)	51 (72.9)	54 (75.0)	45 (60)	
Oily	51 (23.5)	16 (22.9)	15 (20.8)	20 (26.7)	
Sensitive	03 (1.4)	00 (0.0)	02 (2.8)	01 (1.3)	
**Comorbidity**†					0.295
Yes	133 (61.3)	47 (67.1)	45 (62.5)	41 (54.7)	
No	84 (38.7)	23 (32.9)	27 (37.5)	34 (45.3)	
**Sweating** (days)	0.8±1.6	0.8±1.6	0.6±1.0	1.0±2.0	0.663
**Oily skin** (days)	4.1±4.2	4.3±3.8	3.8±4.0	4.1±4.6	0.556
**Follow-up time** (days)	8.8±6.2	8.1±4.1	9.3±7.6	8.8±6.3	0.991
**Fever** (days)	1.4±3.0	0.6±1.3^a^	2.6±4.7^b^	0.9±1.4^a^	**<0.001**
**Corticosteroid use** (days) ‡	3.7±4.6	4.3±4.2	3.3±4.3	3.4±5.1	**0.019**
**RASS score**	-3.7±1.8	-3.9±1.7	-3.3±2.0	-3.9±1.6	**0.024**
**Use of Mechanical Ventilation** (days)	11.1±7.8	9.9±6.4	12.2±9.2	11.2±7.4	0.464
No	13 (6.0)	06 (8.6)	03 (4.2)	04 (5.3)	0.519
Yes	204 (94.0)	64 (91.4)	69 (95.8)	71 (94.7)	
**Anasarca** (days)	5.7±5.9	4.4±4.4	6.2±6.7	6.4±6.2	0.221
**Braden scale score**	10.4±1.4	10.2±1.3	10.6±1.5	10.5±1.3	0.355
**NUTRIC Score**	4.1±1.7	4.0±1.6	4.0±1.5	4.3±1.9	0.499
**Time in the ICU** (days)	12.3±7.8	11.3±6.0	13.2±9.0	12.4±8.1	0.746
**NET usage time** (days)	12.5±8.2	12.1±7.6	12.9±8.9	12.5±8.2	0.891
**Reason for leaving the study**					**0.003**
Discharged	86 (39.6)	36 (51.4)	30 (41.7)	20 (26.7)	
Death	69 (31.8)	22 (31.4)	17 (23.6)	30 (40.0)	
Taken off NET	28 (12.9)	09 (12.9)	07 (9.7)	12 (16.0)	
Transferred	34 (15.7)	03 (4.3)	18 (25.0)	13 (17.3)	

**Notes:** M: Mean; SD: Standard Deviation; RASS: Richmond Agitation and Sedation Scale; ICU: Intensive Care Unit; NET: Nasoenteral Tube. *P-values referring to global comparisons between groups: Kruskal-Wallis for continuous variables and Pearson’s Chi-squared for categorical variables. Different letters on the same line indicate a statistically significant difference (*p* < 0.05) in post-hoc multiple comparisons (Dunn for continuous variables; Bonferroni for categorical). Equal letters indicate no statistical difference between groups

† Several comorbidities were monitored, as well as the reason for admission to the ICU, with no statistically significant difference between the groups.

‡ The use of anticoagulants, chemotherapy and sedatives was also monitored, with no statistically significant difference between the groups.

### 3.2 Primary and secondary outcomes

As shown in Table 2, the most common types of medical adhesive-related skin injury were skin peeling and irritant contact dermatitis. Medical device-related pressure injuries were predominantly deep tissue and unclassifiable injuries, while nasoenteral tube detachment was the most frequent tube-related event, followed by displacement and inadvertent removal. There was a statistically significant association between the type of securement and medical adhesive-related skin injury incidence, with a lower frequency in the Hydrocolloid group compared to the Acrylate and Control groups, as well as between the securement type and nasoenteral tube detachment, being more frequent in the Hydrocolloid group and less frequent in the Control group. Differences were also found between the groups regarding outcome in the intensive care unit and hospital, with a higher death rate in the control group and lower in the acrylate group.

**Table 2:** Primary and secondary outcomes in the intervention and control groups for different nasoenteral tube securement methods.

Variables	Total (*n*=217) M±SD or n (%)	Group, M±SD or *n* (%)			p*
		Hydrocolloid (*n*=70)	Acrylate (*n*=72)	Control (*n*=75)	
**MARSI**					**<0.001**
No	158 (72.8)	65 (92.9)	44 (61.1)	49 (65.3)	
Yes	59 (27.2)	05 (7.1)^a^	28 (38.9)^b^	26 (34.7)^b^	
**MARSI Classification** (*n*=59)					0.687
Allergic dermatitis	03 (5.1)	00 (0.0)	02 (7.1)	01 (3.8)	
Irritant contact dermatitis	20 (33.9)	02 (40.0)	11 (39.3)	07 (26.9)	
Skin peeling	22 (37.3)	03 (60.0)	09 (32.1)	10 (38.5)	
Tension or bullous injury	14 (23.7)	00 (0.0)	06 (21.4)	08 (30.8)	
**Time to MARSI** (d)	6.0 (4.4)	5.2 (2.9)	6.4 (4.9)	5.8(4.1)	0.920
**MDRPI**					0.217
No	191 (88)	65 (92.9)	60 (83.3)	66 (88)	
Yes	26 (12)	05 (7.1)	12 (16.7)	09 (12)	
**MDRPI Classification** (*n*=26)					0.826
Stage 1	04 (15.4)	00 (0.0)	03 (23.1)	01 (11.1)	
Stage 2	05 (19.2)	01 (25.0)	01 (7.7)	03 (33.3)	
Stage 3	01 (3.8)	00 (0.0)	01 (7.7)	00 (0.0)	
Deep Tissue Injury	10 (38.5)	02 (50.0)	05 (38.5)	03 (33.3)	
Unclassifiable	06 (23.1)	01 (25.0)	03 (23.1)	02 (22.2)	
**Time to MDRPI** (d)	7.6 (4.8)	6.8 (3.1)	8.5 (5.4)	6.8 (4.9)	0.650
**MMPI**					0.790
No	192 (88.5)	62 (88.6)	65 (90.3)	65 (86.7)	
Yes	25 (11.5)	08 (11.4)	07 (9.7)	10 (13.3)	
**Time to MMPI** (d)	5.8 (3.4)	6.3 (4.7)	6.6 (3.2)	4.6(1.8)	0.437
**Adhesive detachment**					**0.025**
No	72 (33.2)	16 (22.9)	23 (31.9)	33 (44)	
Yes	145 (66.8)	54 (77.1)^a^	49 (68.1)^a,b^	42 (56)^b^	
**Time to 1st adhesive detachment** (d)^†^	4.1 (2.8)	3.6 (2.1)	3.8 (2.3)	5.2 (3.8)	0.194
**NET displacement**					0.450
No	135 (62.2)	41 (58.6)	49 (68.1)	45 (60)	
Yes	82 (37.8)	29 (41.4)	23 (31.9)	30 (40)	
**Time to 1st displacement** (d) ^†^	6.5 (4.1)	5.2 (3.1)	7.7 (4.4)	6.7 (4.4)	0.074
**Inadvertent NET removal**					0.509
No	158 (72.8)	49 (70)	56 (77.8)	53 (70.7)	
Yes	59 (27.2)	21 (30%)	16 (22.2)	22 (29.3)	
**Time to extubation** (d)	8.4 (4.9)	6.4 (3.0)	9.4 (5.6)	9.4 (5.2)	0.078
**Interruption of enteral feeding**					0.437
No	160 (73.7)	50 (71.4)	57 (79.2)	53 (70.7)	
Yes	57 (26.3)	20 (28.6)	15 (20.8)	22 (29.3)	
**Time to interruption** (h)	1.8±3.8	2.0±4.1	1.5±3.6	1.9±3.6	0.507
**ICU outcome**					**0.003**
Discharge	89 (41)	38 (54.3)^a^	28 (38.9)^a,b^	23 (30.7)^b^	
Death	70 (32.3)	22 (31.4)	17 (23.6)	31 (41.3)	
Transfer	58 (26.7)	10 (14.3)^a^	27 (37.5)^b^	21 (28)^a,b^	
**Hospital outcome**					**0.001**
Discharge	88 (40.6)	35 (50)	26 (36.1)	27 (36)	
Death	78 (35.9)	26 (37.1)^a,b^	18 (25)^a^	34 (45.3)^b^	
Transfer	51 (23.5)	09 (12.9)^a^	28 (38.90^b^)	14 (18.7)^a^	

**Notes:** M: Mean: SD: Standard Deviation; MARSI: medical adhesive-related skin injury; NET: Nasoenteral Tube; MDRPI: medical device-related pressure injuries; MMPI: mucosal membrane pressure injury. *p-values referring to overall comparisons between groups: Kruskal-Wallis for continuous variables and Pearson’s Chi-squared for categorical variables. Different letters on the same line indicate a statistically significant difference (*p* < 0.05) in post-hoc multiple comparisons (Dunn for continuous variables; Bonferroni for categorical). Equal letters indicate no statistical difference between groups. ^†^Days from follow-up initiation to the first occurrence of the event, irrespective of the scheduled replacement interval.

### 3.3 Adjusted analyses

The adjusted analysis using GLMM revealed no statistically significant differences between the groups regarding the outcomes of tube retention, which includes inadvertent removal and displacement without extubation. On the other hand, there was a statistically significant difference between the groups for the detachment outcome. The Hydrocolloid group presented a higher risk of detachment when compared to the control group, while the acrylate group did not differ significantly from the control (Table 3).

**Table 3:** Adjusted comparative analysis of efficacy and safety outcomes between Nasoenteral Tube securement devices.

Variables	Group M±SD or *n* (%)			*p*	RR or Difference of Means (95% CI)		
	Ctrl	Acry	Hyd		Acry vs Ctrl	Hyd vs Ctrl	Acry vs Hyd
**Inadvertent removal**	22 (29.3)	16 (22.2)	21(30)	0.517	0.68 (0.32-1.44)	1.00 (0.49-2.06)	0.68 (0.32-1.45)
**Displacement without extubation**	30 (40)	23 (31.9)	29 (41.4)	0.528	0.71 (0.36-1.41)	1.02 (0.52-1.99)	0.70 (0.35-1.40)
**Detachment**	42 (56)	49 (68.1)	54 (77.1)	**0.044**	1.62 (0.81-3.21)	2.52 (1.21-5.24)	0.64 (0.30-1.37)
**MARSI**	26 (34.7)	28 (38.9)	05 (7.1)	**<0.001**	1.17 (0.60-2.31)	0.14 (0.05-0.39)	8.40 (2.99-23.58)
**MMPI**	10 (13.3)	07 (9.7)	08 (11.4)	0.818	0.72 (0.25-2.01)	0.89 (0.33-2.41)	0.81 (0.27-2.38)
**Duration of dietary interruption** (h)	1.9±3.6	1.5±3.6	2.0±4.1	0.809	-0.39 (-1.59; 0.81)	-0.10 (-1.31; 1.11)	-0.29 (-1.51; 0.94)

**Notes:** M: Mean: SD: Standard Deviation; RR: Relative risk. CI: Confidence Interval; Ctrl: Control; Acry: Acrylate Hyd: Hydrocolloid; MARSI: medical adhesive-related skin injury; MMPI: mucosal membrane pressure injury. Inferential analysis was performed using Generalized Linear Mixed Models to adjust for intra-cluster data dependence. The models included the intervention group and study period as fixed effects, and the participating center (Hospital) as a random effect (intercept). The effect estimate presented for binary variables is the Risk Ratio.

Regarding skin safety, the Hydrocolloid device demonstrated a statistically significant protective effect compared to the control, with an 86% reduction in the risk of medical adhesive-related skin injury. The Acrylate group did not show a statistically significant difference in relation to the control for this outcome. When comparing dedicated devices, the Acrylate group presented a significantly higher medical adhesive-related skin injury risk compared to the Hydrocolloid group.

### 3.4 Clinical impact measures

The evaluation of clinical impact indicators did not demonstrate a statistically significant absolute risk reduction for the outcomes of inadvertent removal or displacement of the tube with the use of dedicated devices compared to control. The hydrocolloid group showed an absolute increase in the risk of securement detachment of 19.9%. The acrylate group did not show a statistically significant difference for this outcome. In contrast, the Hydrocolloid groups showed an absolute risk reduction of 28.0% for the medical adhesive-related skin injury outcome, with an NNT estimated at 3.6, which indicates that approximately four patients would need to be treated with this device to prevent an episode of skin injury. The results are detailed in Supplementary Material, Table S2.

### 3.5 Survival analysis

Survival analysis for time to detachment (Supplementary Material, Figure S3A) showed a statistically significant difference between the groups (log-rank *p*=0.0047). Survival curves indicated lower securement durability in the Hydrocolloid group, with a more pronounced decline over time, while the Control group showed the most stable retention curve.

Multiple comparisons confirmed a significant difference between Hydrocolloid and Control. In the Cox regression analysis with mixed effects, the Hydrocolloid group was associated with an increased instantaneous risk of detachment compared to Control. The survival analysis (Supplementary Material, Figure S3) did not show a statistically significant difference between the groups (log-rank *p*=0.37) for the outcome of time to tube displacement. The curves overlapped over the follow-up period. In the adjusted analysis, dedicated devices did not significantly reduce the risk of displacement compared to the control.

## 4 Discussion

We demonstrated that dedicated securement technologies did not show superiority over manufactured securement methods regarding the prevention of nasoenteral tube displacement or inadvertent removal. However, statistically significant differences were in securement durability and skin safety. The hydrocolloid-based strategy was associated with more frequent securement detachment but substantially reduced medical adhesive-related skin injury. Importantly, the increased detachment did not translate into higher rates of tube displacement, inadvertent removal, or interruption of enteral nutrition, indicating a trade-off between securement durability and skin integrity rather than between tube stability and skin safety.

The higher risk of detachment observed in the hydrocolloid group is consistent with the properties of this adhesive technology. Hydrocolloid dressings contain hydrophilic components that absorb fluid; under physiological conditions, progressive swelling, dissolution, and exudation of these components can alter the surface properties of the material and reduce adhesive strength, ultimately leading to detachment (Kong et al., 2020). This mechanism is clinically relevant because excessive exudate, mucous secretions, or sweat may saturate hydrocolloid dressings and require earlier replacement (Chen et al., 2020), while frequent nasoenteral tube manipulation and patient mobilization may further challenge securement durability (Chen et al., 2026). The longer scheduled replacement interval may also have increased the opportunity for adhesive degradation and detachment over time, and this effect cannot be fully separated from the material properties of the hydrocolloid device.

The performance of an adhesive at the skin interface depends on multiple interacting properties, including adhesive strength, moisture management, occlusion, peel behavior, viscoelastic characteristics, and duration of wear (Dyson et al., 2024). Thus, securement durability and skin protection should be considered related but distinct dimensions of clinical performance. Although the hydrocolloid strategy showed lower securement durability, this did not translate into higher rates of tube displacement, inadvertent removal, or interruption of enteral nutrition. Accordingly, the increased detachment observed with the hydrocolloid strategy appears to represent primarily a durability limitation, with a greater need for monitoring and securement replacement, rather than evidence of reduced enteral access safety.

The lower incidence of medical adhesive-related skin injury observed with the hydrocolloid strategy should be interpreted as an effect of the overall securement strategy rather than as evidence that hydrocolloid adhesives are intrinsically less traumatic to the skin. Hydrocolloid dressings can provide a protective interface and have been associated with reduced device-related pressure injury and prolonged skin integrity (Chen et al., 2020). However, their cutaneous effects are formulation- and context-dependent; a hydrocolloid adhesive has been associated with greater peel adhesion, stratum corneum protein removal, discomfort, erythema, and post-removal impairment of skin barrier function than a silicone comparator (Dyson et al., 2024). Repeated adhesive application and removal may also contribute to skin injury (Dyson et al., 2024). In our trial, the 7-day replacement interval resulted in fewer planned removal and reapplication episodes than the 24-hour schedule used in the control and acrylate groups. Therefore, the protective effect observed in this trial should be interpreted as the effect of the overall securement strategy rather than of adhesive composition alone.

Although important differences in securement durability and medical adhesive-related skin injury occurrence were observed, the dedicated technologies did not significantly reduce nasoenteral tube displacement or inadvertent removal. These events are multifactorial and cannot be attributed solely to the adhesive properties of the securement technology. Patient- and care-related factors, including altered cognitive status, awakening from sedation, mobilization, and manual handling, as well as age, neurological history, and level of consciousness, may also contribute to tube displacement or inadvertent removal (Anziliero & Beghetto, 2022; Frota et al., 2025). These findings reinforce that nasoenteral tube stability depends on the interaction between the securement strategy, patient characteristics, and care processes rather than on adhesive performance alone.

This interpretation aligns with implementation evidence showing a reduction in nasoenteral tube-related complications when securement technologies are integrated into multifaceted care strategies, such as structured staff training, standardized protocols, clinical decision support tools, and the reorganization of care processes, rather than being used as isolated interventions (Ribeiro et al., 2025). In this light, the present study complements existing evidence by demonstrating that, even with rigorous standardization of the securement technique and thorough training and calibration of the research team, securement technology alone was insufficient to alter key mechanical outcomes; this reinforces the idea that its effectiveness depends on its interaction with the care context.

The primary clinical implication of this study is that the choice of securement technology should not be guided solely by adhesive strength. No single technology outperformed the others across all evaluated outcomes, highlighting a trade-off between securement durability and preservation of skin integrity. Therefore, technology selection should consider the patient’s risk profile, including skin fragility, risk of medical adhesive-related skin injury, likelihood of nasoenteral tube displacement, intensity of device manipulation, and the staff’s ability to detect and replace failed securement promptly.

This perspective is consistent with current evidence indicating that the selection of adhesive technologies should balance effective securement with preservation of skin integrity (Kong et al., 2020; Dyson et al., 2024). Viewed in this light, the clinical objective shifts from identifying a universally superior device to selecting the most appropriate securement strategy for each clinical context.

### 4.1 Implications for practice, education, and research

The results also have significant implications for nursing management, education, and research. From a management perspective, they reinforce the need for standardized institutional protocols regarding the selection, application, inspection, and removal of adhesive technologies, as well as specific indicators related to nasoenteral tube stability and medical adhesive-related skin injury occurrence. Regarding professional training, they highlight the importance of systematic education on securement technologies, skin injury prevention, and the continuous monitoring of medical devices. For research, the findings indicate a need for multicenter studies that incorporate cost-effectiveness analyses and develop new adhesive technologies capable of combining greater securement durability with a lower potential for skin trauma, thereby reducing the current trade-off between mechanical stability and skin safety.

### 4.2 Strengths

The pragmatic design randomized by clusters with crossover increases external validity and reduces contamination between interventions, the standardization of securement technologies through standard operating procedures based on manufacturers’ recommendations, and a methodological validation study for manufactured securement (Vilalba, 2025) are among the main strengths of this study, in addition to the training and calibration of researchers. Additionally, the use of mixed statistical models and survival analyses strengthened the robustness of the estimates when considering the correlated structure of the data.

### 4.3 Limitations

Several limitations should be considered. Complete blinding of the care team was not feasible because the securement technologies were visually distinguishable. Variations inherent to intensive care, between-group differences in some clinical characteristics, daily outcome assessments, and unequal follow-up resulting from discharge, transfer, or death may also have influenced the findings, although these factors were addressed in the study design and statistical analyses. Sequential Organ Failure Assessment (SOFA) and Acute Physiology and Chronic Health Evaluation (APACHE) scores were unavailable for a substantial proportion of participants and were, therefore, not included in baseline comparisons. Consequently, residual differences in baseline illness severity across intensive care unit-periods cannot be excluded and may have contributed to the observed differences in intensive care unit and hospital outcomes. In addition, scheduled replacement intervals differed across securement strategies according to manufacturers’ recommendations. Consequently, differences in skin injury and securement durability may reflect both adhesive properties and different frequencies of removal and reapplication, and these effects cannot be fully disentangled. Finally, although the analyses accounted for clustering through hospital-level random effects and adjustment for the study period, only three intensive care units were randomized. This small number of clusters may have reduced the precision of cluster-level estimates and may limit inference at the cluster level.

## 5 Conclusions

Dedicated securement technologies did not demonstrate superiority over manufactured securement in preventing nasoenteral tube displacement or inadvertent removal. The hydrocolloid-based strategy substantially reduced medical adhesive-related skin injury but showed lower securement durability, with more frequent replacement. Importantly, this increased detachment did not translate into greater tube displacement, inadvertent removal, or interruption of enteral nutrition. From these findings, we support balancing securement durability and skin integrity when selecting a securement strategy.

## Declaration of generative artificial intelligence

During the preparation of this work, the authors used ChatGPT (OpenAI) to support language editing and improve the clarity, organization, and readability of the manuscript. After using this tool, the authors reviewed and edited the content as needed and take full responsibility for the content of the publication.

## Clinical trial registration

Brazilian Registry of Clinical Trials (ReBEC): RBR-10hr8nzd.

## Ethics approval

The study was approved by the Research Ethics Committees of all participating institutions. Written informed consent was obtained from all participants or their legal representatives before enrolment in the study.

## Ethics declaration

Written informed consent to take part in the study and to publish the article has been obtained from all participants or their legal representatives. The privacy rights of participants have been observed.

This study was performed in compliance with relevant laws, regulatory frameworks and guidelines where the research took place. This study was conducted in accordance with The study was conducted in accordance with the World Medical Association Declaration of Helsinki (2024 revision) and the ethical principles established by the Brazilian National Health Council Resolution No. 466/2012. This study was approved by the Research Ethics Committee of Maria Aparecida Pedrossian University Hospital. (Approval No. 6.687.415)

The results of this clinical trial and any associated work have been posted in a registry. This clinical trial was registered with number RBR-10hr8nzd (Brazilian Registry of Clinical Trials).

## Funding

This study was funded by the *Conselho Nacional de Desenvolvimento Científico e Tecnológico* (CNPq) under Call CNPq/MCTI No. 10/2023 – Universal, Grant No. 405577/2023-0 (Protocol No. 3711000129304867). This study was partly financed by the *Coordenação de Aperfeiçoamento de Pessoal de Nível Superior - Brasil* (CAPES) - Finance Code 001. Additional institutional support was provided by the Federal University of Mato Grosso do Sul (UFMS/MEC), Brazil.

## CRediT authorship contribution statement

**Alan Maique Ribeiro Fernandes da Costa:** Writing – review & editing, Writing – original draft, Visualization, Validation, Supervision, Project administration, Methodology, Investigation, Formal analysis, Data curation, Conceptualization. **Oleci Pereira Frota:** Writing – review & editing, Writing – original draft, Visualization, Validation, Supervision, Software, Resources, Project administration, Methodology, Investigation, Funding acquisition, Formal analysis, Data curation, Conceptualization. **Marcos Antonio Ferreira Junior:** Writing – review & editing, Visualization, Validation, Supervision, Methodology, Formal analysis, Data curation, Conceptualization. **Daniele Vieira Dantas:** Writing – review & editing, Writing – original draft, Visualization, Validation, Methodology, Investigation, Formal analysis, Data curation. **Maria Luiza Berti de Oliveira:** Writing – review & editing, Writing – original draft, Visualization, Validation, Methodology, Investigation, Data curation. **Milena Agnelo Santos Chaves:** Writing – review & editing, Writing – original draft, Visualization, Validation, Supervision, Methodology, Investigation, Formal analysis, Data curation. **Danielle Neris Ferreira:** Writing – review & editing, Writing – original draft, Visualization, Validation, Methodology, Investigation, Funding acquisition, Formal analysis, Data curation, Conceptualization. **Elaine Aparecida Rocha Domingues:** Writing – review & editing, Writing – original draft, Visualization, Validation, Methodology, Investigation, Formal analysis, Data curation.

## Declaration of competing interest

The authors declare that they have no known competing financial interests or personal relationships that could have appeared to influence the work reported in this paper.

## References

[bib0001] Aeberhardt L.E., Bains V.K., Desai S. (2022). Safety and effectiveness of the nasal bridle securement device to retain feeding tubes in adult patients in the intensive care unit: an observational study. Nutr. Clin. Pract..

[bib0002] Anziliero F., Beghetto M.G. (2022). Incidence and risk factors for nasoenteral tube-related mechanical complications in a double cohort. Nutr. Hosp..

[bib0003] Chen J., Chen J., Yang J., Chen Y., Liang Y., Lin Y. (2020). Investigating the efficacy of hydrocolloid dressing for preventing nasotracheal tube-related pressure injury in the PICU. Pediatr. Crit. Care Med..

[bib0004] Chen J., Yu P., Liu L., Chen M., Yuan X., Xu Y. (2026). Incidence and influencing factors of medical adhesive-related skin injury in critically ill patients: a systematic review and meta-analysis. Aust. Crit. Care.

[bib0005] Dyson E., Sikkink S., Nocita D., Twigg P., Westgate G., Swift T. (2024). Evaluating the irritant factors of silicone and hydrocolloid skin contact adhesives using trans-epidermal water loss, protein stripping, erythema, and ease of removal. ACS Appl. Bio Mater..

[bib0006] Haesler E., European Pressure Ulcer Advisory Panel, National Pressure Injury Advisory Panel, Pan Pacific Pressure Injury Alliance (2019). The International Guideline.

[bib0007] Frota O.P., Paula F.M., Ferreira D.N., Ferreira-Júnior M.A., Domingues E.A.R., Ruiz J.S. (2025). Incidence, characteristics and influencing factors of medical adhesive-related skin injury in the indwelling urinary catheter attachment region in critically ill patients: a longitudinal study. Nurs. Crit. Care.

[bib0008] Kong, D., Zhang, Q., You, J., Cheng, Y., Hong, C., Chen, Z., et al., 2020. Adhesion loss mechanism based on carboxymethyl cellulose-filled hydrocolloid dressings in physiological wounds environment. Carbohydrate Polymers. 235:115953. doi: 10.1016/j.carbpol.2020.115953. 10.1016/j.carbpol.2020.115953 32122489

[bib0009] McNichol L., Lund C., Rosen T., Gray M. (2013). Medical adhesives and patient safety: state of the science: consensus statements for the assessment, prevention, and treatment of adhesive-related skin injuries. J. Wound Ostomy Cont. Nurs..

[bib0010] Motta A.P.G., Rigobello M.C.G., Silveira R.C.C.P., Gimenes F.R.E. (2021). Nasogastric/nasoenteric tube-related adverse events: an integrative review. Rev. Lat.-Am. Enferm..

[bib0011] Ribeiro F.A., Sodré da Costa L.S., Pedroso A.C., de P., Nogueira P.B., Brandi S., Toledo D.O. (2025). Evaluating multifaceted strategies to prevent nasoenteral tube complications and achieve significant cost savings in critically ill patients: the ENHANCE-CRIT trial. BMJ Open Qual..

[bib0012] Vilalba A.B.C. (2025). https://repositorio.ufms.br/handle/123456789/12830.

