# Supplementary material for: Effectiveness of three nasoenteral tube securement technologies in critically ill adults: A pragmatic cluster-randomized crossover trial
Source: Int J Nurs Stud Adv. 2026 Oct 27;11:100729. doi: 10.1016/j.ijnsa.2026.100729 (PMC13756790; doi:10.1016/j.ijnsa.2026.100729)
Supplement: Supplementary file 1 [file mmc1.docx]

S**upplementary figure**

**Supplementary figure S1**. Securement technologies evaluated in the trial: (A) manually prepared acrylic adhesive tape (control); (B) dedicated acrylic-based securement device; and (C) dedicated hydrocolloid-based securement device.

| 🅐 | 🅑 | 🅒 |
| --- | --- | --- |
