# Supplementary material for: Effectiveness of three nasoenteral tube securement technologies in critically ill adults: A pragmatic cluster-randomized crossover trial
Source: Int J Nurs Stud Adv. 2026 Oct 27;11:100729. doi: 10.1016/j.ijnsa.2026.100729 (PMC13756790; doi:10.1016/j.ijnsa.2026.100729)
Supplement: Supplementary file 2 [file mmc2.docx]

S**upplementary figure**

**Supplementary figure S2.** Kaplan-Meier survival curves for (A) time until adhesion loss of the NET; and (B) time for the NET to remain free from displacement according to the securement device

**A**

**Note A:** The horizontal axis represents the days that elapsed until the adhesive came off (event). The overall p-value refers to the Log-Rank test (p=0.0047).

**B**

**Note B:** The horizontal axis represents the days elapsed from securement to the event (displacement) or censoring. Vertical marks (+) on the lines indicate censored data (patients who were discharged, died or ended the collection period without showing tube displacement). The p-value refers to the Log-Rank test for overall comparison between the three curves.
