# Supplementary material for: Effectiveness of three nasoenteral tube securement technologies in critically ill adults: A pragmatic cluster-randomized crossover trial
Source: Int J Nurs Stud Adv. 2026 Oct 27;11:100729. doi: 10.1016/j.ijnsa.2026.100729 (PMC13756790; doi:10.1016/j.ijnsa.2026.100729)
Supplement: Supplementary file 3 [file mmc3.docx]

S**upplementary table**

**Supplementary Table S1.** Clinical impact indicators of Nasoenteral Tube securement devices

| **Variable** | **Comparison** | **Relative Risk**  **[95%CI]** | **Absolute Risk Reduction (ARR) [95%CI]** | **NNT**  **[95%CI]** |
| --- | --- | --- | --- | --- |
| **Inadvertent removal** | Acrylate vs Control | -0.07 [-0.22; 0.07] | 7.5% [-6.8; 21.6] | NS |
|  | Hydrocolloid vs Control | 0.00 [-0.15; 0.15] | -0.1% [-15.0;14.9] | NS |
| **Displacement** | Acrylate vs Control | -0.08 [-0.23; 0.08] | 7.8% [-7.9; 23.5] | NS |
|  | Hydrocolloid vs Control | 0.00 [-0.16; 0.17] | -0.4% [-16.5; 15.7] | NS |
| **Detachment** | Acrylate vs Control | 0.11 [-0.05; 0.27] | -11.1% [-26.9; 4.6] | NS |
|  | Hydrocolloid vs Control | 0.20 [0.05; 0.35] | -19.9% [35.0; 4.8] | -5.0 [-20.9; 2.9] |
| **MARSI** | Acrylate vs Control | 0.04 [-0.12; 0.19] | -3.8% [-19.5; 12.0] | NS |
|  | Hydrocolloid vs Control | -0.28 [-0.40; -0.16] | 28.0% [15.6; 40.4] | 3.6 [2.5; 6.4] |
| **MMPI** | Acrylate vs Control | -0.03 [-0.13; 0.07] | 3.3% [-6.8; 13.3] | NS |
|  | Hydrocolloid vs Control | -0.01 [-0.12; 0.09] | 1.3% [-9.3; 11.9] | NS |

**General note**: Estimates are based on marginal probabilities derived from Generalized Linear Mixed Models (GLMM) adjusted by cluster and time. The ARR corresponds to the difference between the risk in the control group and the risk in the intervention group (subtraction); Negative values indicate increased risk. The NNT was calculated as 1/ARR: positive values indicate benefit (number needed to treat to prevent an event) and negative values indicate harm (Number Needed to Harm); vs: versus*.*

**Specific notes:** 95%CI: 95% Confidence Interval; RR: Relative Risk; NNT: Number Needed to Treat; NS: Not Significant MARSI: medical adhesive-related skin injury; MMPI: mucosal membrane pressure injury.
